# Identification and Characterization of a Potential Antibiotic Producing Strain of *Pantoea ananatis*

**DOI:** 10.7150/jgen.70066

**Published:** 2022-02-07

**Authors:** Michael J. LaGier, Mark McDaniel, Alyssa Ragner, Amber Castillo

**Affiliations:** Department of Biology, Grand View University, Des Moines, IA, USA.

**Keywords:** *Pantoea*, antibiotic, soil

## Abstract

Antibiotic resistance continues to be a significant public health challenge. Soil bacteria represent a potential source of yet to be discovered antimicrobials. The screening of Iowa (United States) soils yielded the identification of a strain of *Pantoea ananatis* (MMB-1), which displayed an antimicrobial-producing phenotype against a bacterium (*Bacillus subtilis*) representative of Gram-positive bacteria. Crude, organic, extracts of MMB-1 retained the anti-microbial activity. The draft genome of strain MMB-1 contains a total of 4,634,340 bp, and 4,624 protein-encoding genes. Consistent with phenotypic observation, the genome of MMB-1 encodes for a number of putative secondary metabolite biosynthetic gene clusters, including those known to be involved in the production of the antibiotics lankacidin C and bottromycin. This study increases our overall understanding of *Panteoa* as a group, and is also consistent with the notion that members of this genus have significant potential as useful natural product producers.

## Introduction

Members of the Genus Pantoea include at least 35 known species (http://www.bacterio.net), which can be found in a variety of habitats, both terrestrial and aquatic 1. Pantoea are Gram-negative bacteria, rod-shaped, and often produce mucoid colonies when grown on semi-solid media 1. A number of identified species produce antimicrobials, and have been developed into biocontrol products, including Bloomtime Biological, which is used to control apple and pear fire blight 2. Others are thought to contain bioremediation potential, specifically, in the degradation of herbicides 3. Some species of this genus also appear to behave as plant and animal pathogens 1.

The goal of the current study was to screen Iowa soils, close to the Des Moines metropolitan area, for soil bacteria that show the potential to produce antimicrobial substances. The persistence of antibiotic resistance as a public health threat remains; and one way to help lessen this threat, is through the discovery of new, naturally-occurring, antimicrobials. Soil bacteria are known antimicrobial producers 4, and, indeed, a new class of antibiotics was recently discovered from soil 5.

This study identifies a strain of *Pantoea annantis* (MMB-1), isolated from soil, that demonstrates anti-microbial activity. In addition to observing this functional activity for MMB-1, the genome of MMB-1 was also sequenced. The genome of MMB-1 appears to contain genes believed to be involved in the production of secondary metabolites. Antibiotics are products of secondary, bacterial, metabolism 4. Interestingly, strain MMB-1 is not the first *P. annantis* to show antimicrobial activity 1, thus, further highlighting the potential of this species as a source of antimicrobial substances.

## Materials and Methods

### Sample Collection, Strain Isolation and Strain Characterization

The soil from which *Pantoea ananatis* MMB-1 was derived was originally collected in August 2017. Soil was collected 6 cm deep from the surface, in an area surrounding Franklin Cemetery, Northeast of Des Moines (IA, USA), at coordinates 41°42'53.4'' N and 93°22'46.6'' W. The temperature of the soil at the time of collection was 23 °C and the pH was 7.5.

One gram of collected soil was resuspended in 5 mL sterile water, homogenized by vortex, and serial-diluted (sterile water) for standard plate counting. Serial-diluted soil samples were plated on Potato Dextrose Agar (PDA) and incubated at 30 °C for 4 days. Individual colonies were picked and streaked on PDA for isolation. Standard plate counting on PDA estimated the soil sampled contained 3.9 × 10^7^ CFU/gram of soil.

Basic morphological and biochemical tests were carried out against MMB-1, including Gram-staining, indole production, and catalase production, according to standard practice 6. Isolated samples of MMB-1 were stored at -80 °C for long-term use in Nutrient Broth (NB) supplemented with 10% (w/v), sterile, glycerol.

### Screening of MMB-1 for Antimicrobial Activity

An antagonistic activity assay termed “spread-patch” 7, which is a form of competitive plating, was used to determine if MMB-1 can inhibit the growth of other bacteria when co-cultured on the same plate. Competitive plate can be used to screen bacteria for potential production of antimicrobial substances 7. MMB-1 was screened for activity against a bacterium representing Gram-positives (*Bacillus subtilis*, ATCC 6051) and against a bacterium representing Gram-negatives (*Klebsiella pneumoniae*, ATCC 2342) using the procedure as described in 7. In the procedure, a zone of no growth, around the plated “patch” of MMB-1, was interpreted as MMB-1 displaying antagonistic activity against the co-cultured bacterium.

### Crude Extraction of MMB-1 Antagonistic Substances

Antagonistic activity identified by “spread-patch” was confirmed as associated with MMB-1 via crude organic extraction from whole bacteria followed by testing of extracted materials for retained antagonistic activity against bacteria representing Gram-positives. This assay was carried out as described in 7, “Chemical Extraction of Antibiotics” section, using ethyl acetate as the extraction solvent.

### Genomic DNA Isolation

Isolated MMB-1 was growth in NB, and genomic DNA was isolated, according to the included instructions, using a PureLink Genomic DNA Mini Kit (Invitrogen). Integrity of isolated genomic DNA was visualized by agarose gel electrophoresis (0.5% w/v). Isolated DNA was then provided to MR DNA (Shallowater, TX, USA) for whole genome sequencing and assembly.

### Bacterial Genome Sequencing

Sequencing was performed using an Illumina HiSeq system. The concentration of DNA was evaluated using the Qubit® dsDNA HS Assay Kit (Life Technologies). The library was prepared using a Nextera DNA sample preparation kit (Illumina) following the manufacturer's user guide. Following library preparation, the final concentration of the library (5.72 ng/μL) was measured using the Qubit dsDNA HS assay kit (Life Technologies), and the average library size (835 bp) was determined using the Agilent 2100 Bioanalyzer (Agilent Technologies). The libraries were pooled and diluted (to 6.0 pM) and sequenced paired end for 500 cycles (100X coverage). Obtained sequence reads were assembled (MR DNA) into 12 contigs (longest, 1,380,906 bp; N50, 664,687 bp) using NGen (DNASTAR). The resulting genome was annotated using RAST in default settings 8. The software programs antiSMASH 9 and BAGEL 10 were used to identify potential secondary metabolite biosynthetic genes (most recent versions, default settings). This whole genome project was deposited at GenBank under accession (BioProject) PRJNA602096.

## Results and Discussion

### Identification of MMB-1 from Soil

**Figure 1:**
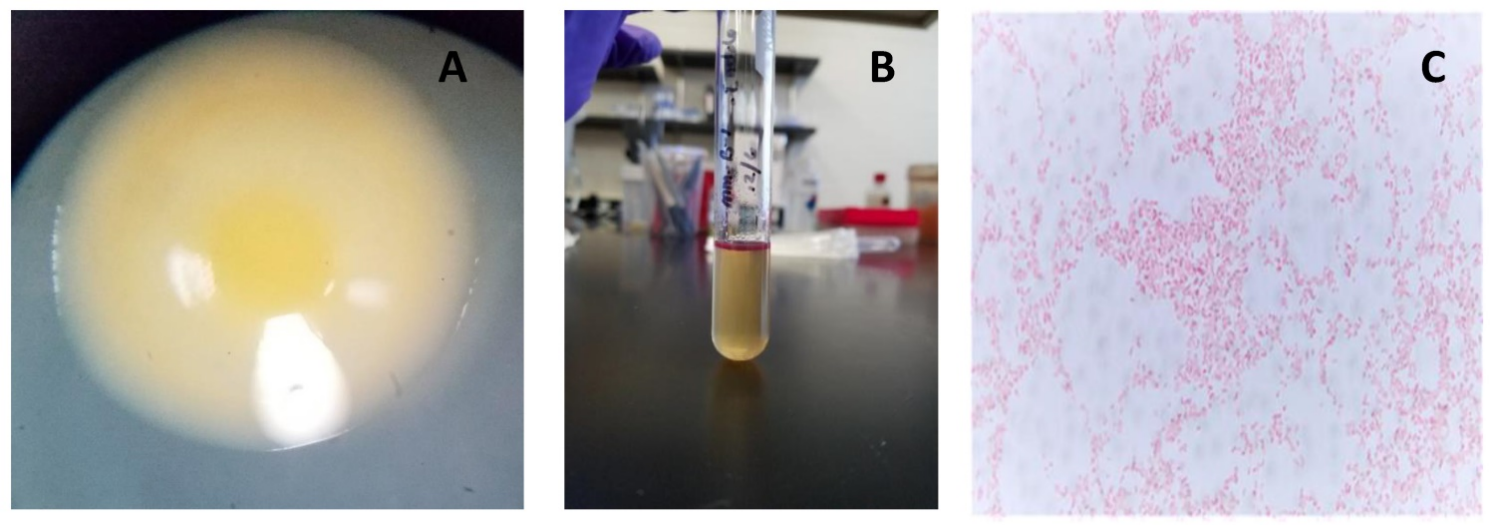
**Observed characteristics of MMB-1. A.** A representative colony of MMB-1 as observed at 40X using a stereomicroscope, showing yellow pigmentation, and a shiny, mucoid-type colony texture. **B.** A positive Indole test was observed (purple-pink color formed at top of test tube) after adding 0.5 mL of Kovac's reagent to MMB-1 grown at 37 °C for 48 hours in 4.0 mL of tryptophan broth. **C.** A representative Gram-stained sample of MMB-1 as observed at 1,000X using brightfield light microscopy. Note the abundance of Gram-negative, single rods.

The strain, designated here as *Pantoea ananatis* MMB-1 displayed characteristics shared among Pantoea including being found in soil, and composed of indole positive, Gram-negative rods, that displayed a mucoid-type morphology when grown on semisolid media (Figure 1). In addition, according to BLASTn analysis (NCBI, default parameters), a representative 16s rRNA gene from the sequenced genome of MMB-1 (Figure 4) showed 99.05% sequence identity to a *P. ananatis* 16s rRNA gene in the NCBI database (strain PA13, accession CP003085.1). Consistent with BLASTn of 16s rRNA, BLASTp (NCBI, default parameters) using a common bacterial housekeeping gene, the chaperonin HSP60 (GroEL) protein, from the genome of MMB-1, showed 98.01% sequence identity to a *P. ananatis* GroEL gene in the NCBI database (strain LMG 20103, accession ADD75568).

### MMB-1 Demonstrates Antimicrobial Activity

**Figure 2:**
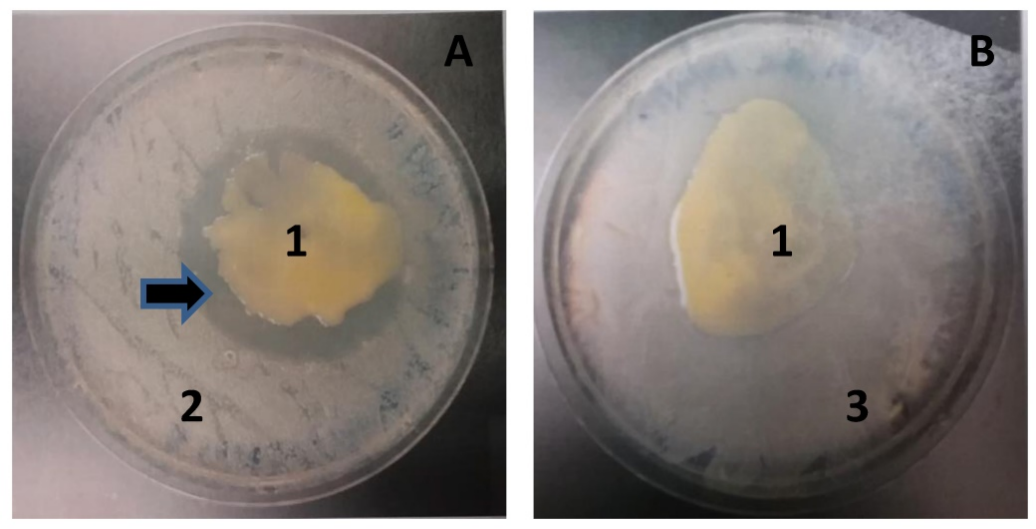
** MMB-1 inhibits the growth of *B. subtilis* when co-cultured. A.** Co-culture of MMB-1 (1) and *B. subtilis* (2). Note the zone of no growth surrounding MMB-1 (arrow). **B.** Co-culture of MMB-1 (1) and *K. pneumoniae* (3). Note the lack of zone of no growth in panel **B.** With both panel **A** and panel **B**, samples were growth for 48 hours at 37 °C on PDA.

Upon co-culturing MMB-1 with the Gram-positive bacterium *B. subtilis*, MMB-1 was observed to produce a zone of no growth surrounding the MMB-1 cells (Figure 2). This suggests that MMB-1 is producing a substance that is inhibiting the co-cultured *B. subtilis*. Such a zone of no growth was not observed when co-culturing MMB-1 with a Gram-negative bacterium, *K. pneumoniae* (Figure 2). Together, this indicates at least one antimicrobial substance is produced by MMB-1 that has potential to inhibit the growth of Gram-positives; but not Gram-negatives. Significantly, several antibiotic-resistant strains, Gram-positive in nature, are of current clinical concern 11.

**Figure 3:**
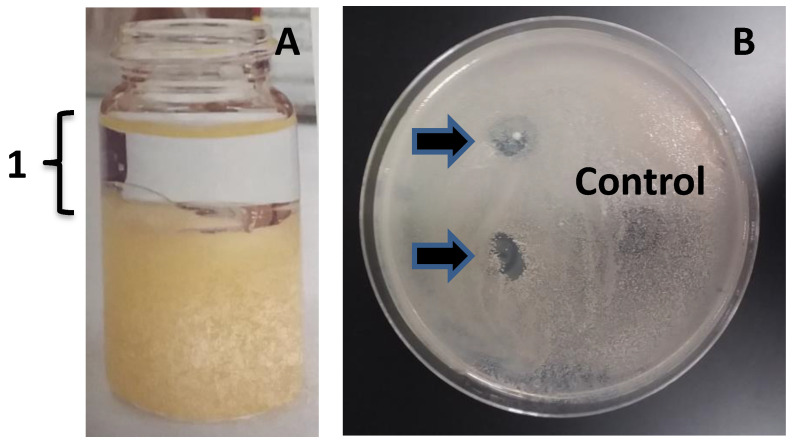
** Ethyl acetate extract of MMB-1 retains the anti-microbial activity. A.** Extract of MMB-1 cells grown on PDA for 48 hours at 37℃ (10 mL glass vial shown). In panel **A**, the organic phase of the extraction is indicated (1). **B.** Testing of organic phase (1, panel **A**). A total of 50 uL (top arrow) and 100 uL (bottom arrow) of organic phase extract were spotted on a PDA plate where a sample of *B. subtilis* was previously spread. Note the zones of no growth where organic extract was added (arrows). Control indicates where 100 uL of ethyl acetate alone was added to the plate. Bacteria were allowed to grow for 48 hours at 37℃ prior to documentation.

To further confirm that MMB-1 is responsible for making a substance, or substances, that can account for the zone of no growth shown in Figure 2, an effort was made to extract organic substances from MMB-1. Specifically, substances soluble in organic solvents (including ethyl acetate, used here), when extracted from whole bacterial cells, can often include antibiotics 7. As shown in Figure 3, when ethyl acetate-based extracts of MMB-1 (from pure cultures of MMB-1) were exposed to *B. subtilis*, the extracts produce zones of no growth that are comparable to the live-organism-based experiment shown in Figure 2. Importantly, as also shown in Figure 3, exposure to the extraction solvent alone, ethyl acetate, alone does not inhibit the growth of *B. subtilis*.

### Genomic Features of MMB-1

**Figure 4:**
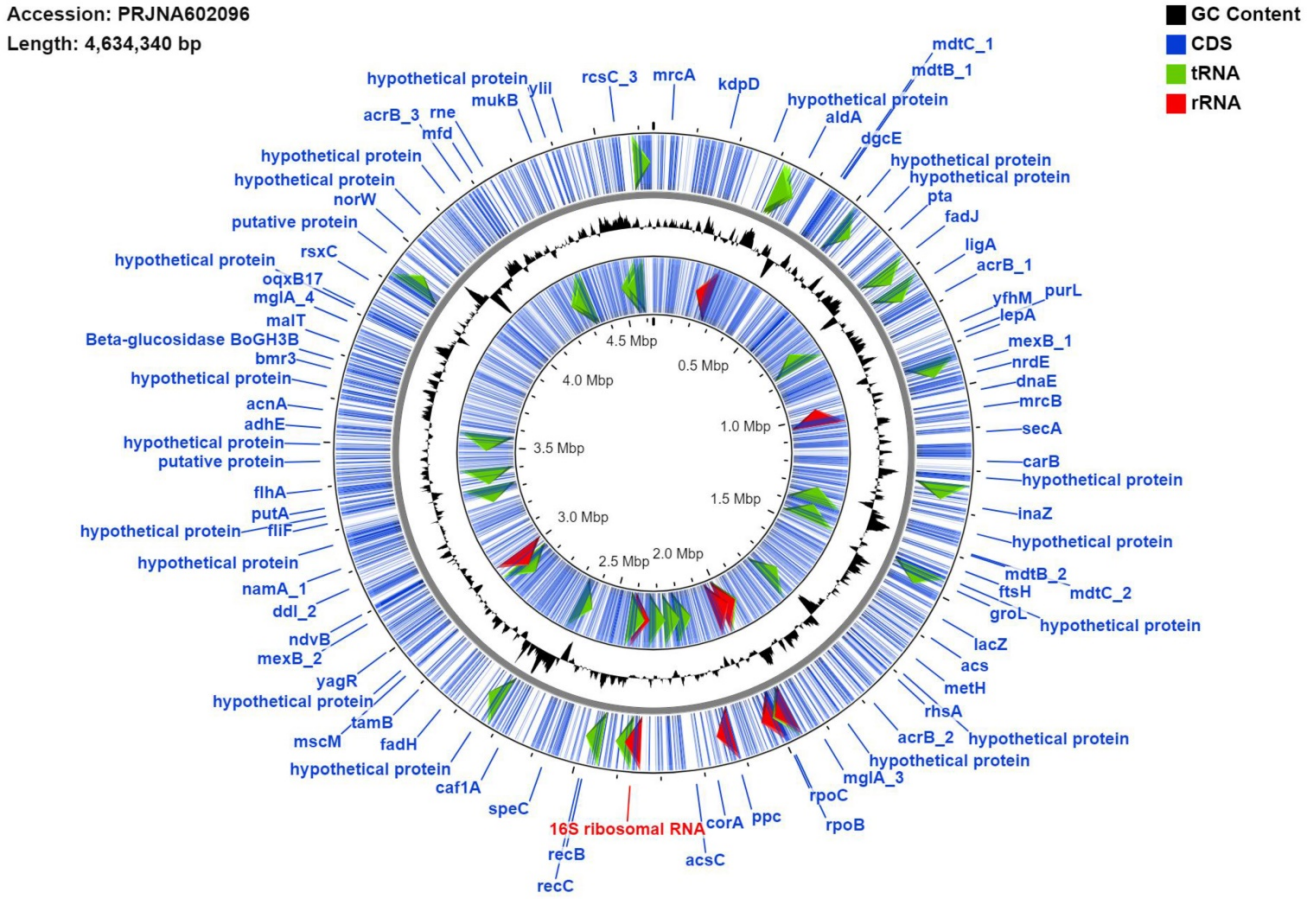
** Genome map of MMB-1.** The map was generated using the default settings of CGView. Blue lines represent identified coding sequences (CDS), red arrows denote identified rRNA sequences, green arrows indicate tRNA sequences. Labels in blue (e.g. groEL, rpoB) are examples of gene names and their genomic locations. The signal graph in black mark GC%. The inner ring labeled with multiple CDS is the reverse strand and the outer ring is the forward. The individual 16S rRNA marked red was used as the BLAST query sequence referenced in the results and discussion (Identification of MMB-1 from Soil).

**Figure 5:**
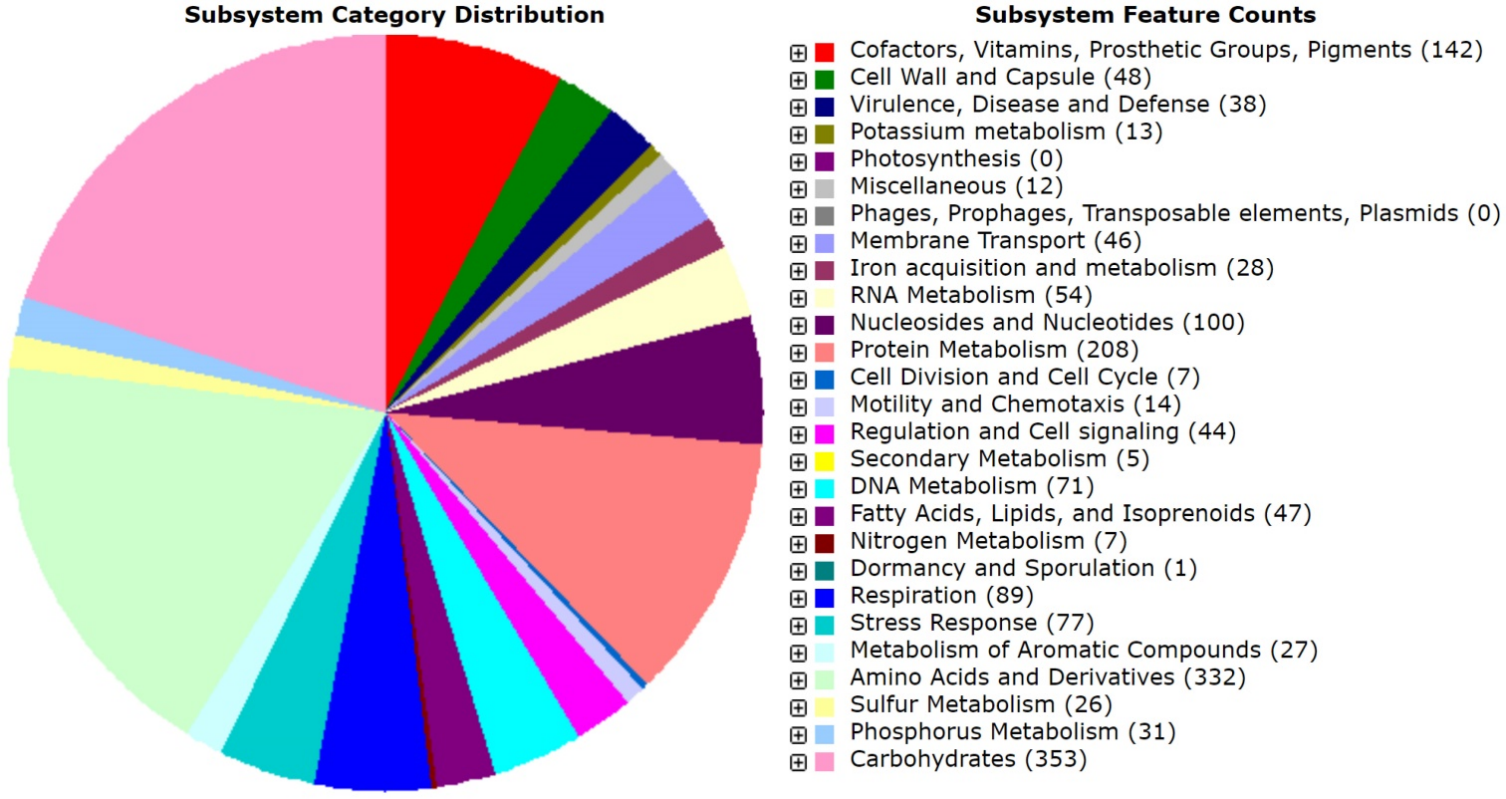
** Subsystem distribution of the MMB-1 genome.** Category distribution is shown, as outputted from RAST. Feature counts the predicted number of genes found by RAST that belong to each functional category. A total of 1,272 genes (28% of total annotated genes) could be placed, stringently, into RAST-based feature count categories, while 3,352 could not.

The draft genome of MMB-1 is 4,634,340 bp. The GC% is 53.6%. RAST (Rapid Annotation using Subsystem Technology) predicts a total of 4,624 genes. A genome map of MMB-1 is shown in Figure 4, as generated using the CGView server 12. The genome size and GC% values fall into ranges observed for other sequenced *Pantoea*, which appear to range from 4.5 - 6.3 million bp in size and 52 - 55 GC% content 13. The total number of genes encoded in the genome of MMB-1 is consistent with additional *P. ananatis* genomes sequenced 13. As anticipated, a large number of subsystem feature counts identified by RAST are responsible for basic life-sustaining needs including 353 related to carbohydrate metabolism, 208 related to protein metabolism, 89 related to cellular respiration, 48 related to the bacterial cell wall, 46 related to membrane transport, and 44 related to signal transduction (Figure 5).

**Figure 6:**
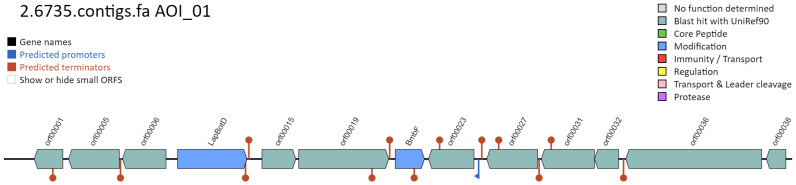
** MMB-1 genome analysis by BAGEL.** According to BAGEL software, the genome of MMB-1 contains a gene cluster with potential to synthesize the bacteriocin (antimicrobial peptide) bottromycin. The graphic (screenshot) shows the organization of genes within this cluster, relative to one another. The genes shaded in blue are locations of genes specifically-linked to bottromycin, BmbF and LapBotD.

Given the potential anti-microbial activity observed by MMB-1 (Figures 2 and 3), an effort was made to identify potential genes that might contribute to such biochemical activity. BAGEL is a web-based server that identifies potential bacteriocin open reading frames from inputted sequences, via the querying of knowledge-based bacteriocin databases 10. Bacteriocins are a diverse group of antimicrobial peptides produced by bacteria 14. According to BAGEL, the MMB-1 genome contains a set of genes that share similarity in sequence, and on the basis of proximity of one another, to a known bacteriocin biosynthetic pathway (Figure 6). Of particular note, this segment of genes (Figure 6), according to BAGEL, contains those related to BmbF and LapBotD (YcaO), both of which are believed to play a role in the production of the bacteriocin bottromycin 15. Interestingly, and consistent with our biochemical data (Figures 2 and 3), bottromycin and bottromycin-like antibiotic peptides appear to show significant antimicrobial activity against Gram-positives 16; although, some also appear to have activity against Gram-negatives.

**Figure 7:**
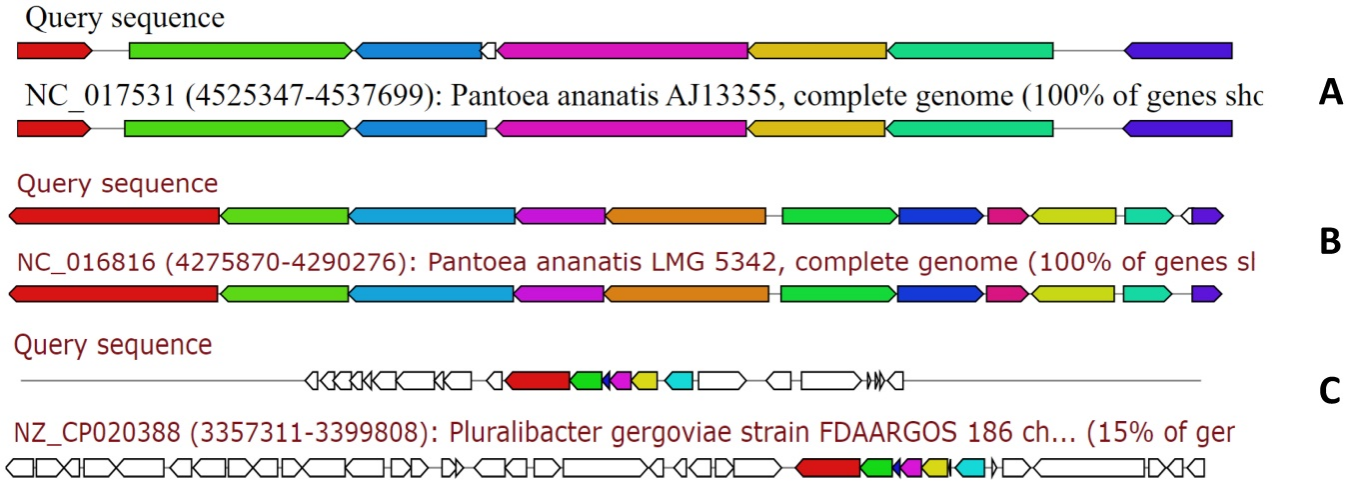
** Examination of the MMB-1 genome using antiSMASH.** Analysis of MMB-1 using antiSMASH identified clusters of genes with potential to synthesize secondary metabolites. Three representative clusters, referenced in the text, are shown (ClusterBlast screenshots). Clusters labeled Query are those from MMB-1, and clusters showing greatest shared organization are placed below MMB-1 gene clusters. **A.** Tentative cluster involved in production of the siderophore aerobactin. **B.** Tentative cluster involved in production of the siderophore desferrioxamine E. **C.** Putative cluster involved in the production of lankacidin-type antibiotics.

The genome of MMB-1 was also examined using antiSMASH. This software is a pipeline for secondary metabolite gene cluster identification. It looks at a variety of secondary metabolism-related genes including non-ribosomal peptides, lantibiotics, and siderophores 9. According to antiSMASH, the genome of MMB-1 contains 23 candidate regions. This value is consistent when compared to other *Pantoea* genomes that have been probed for secondary metabolite gene clusters using antiSMASH; with the range being from 16 to 28 clusters per genome 13, 17. Also consistent with other *Pantoea* genomes scanned by antiSMASH, MMB-1 contains putative biosynthetic gene clusters for the siderophores aerobactin and desferrioxamine E, as well as a tentative cluster for the exopolysaccharide stewartan 13, 17. Interestingly, antiSMASH also identified a cluster for lankacidin C (Figure 7). Lankacidin-type antibiotics are products first identified in soil-dwelling *Streptomyces*, and which show significant antimicrobial activity against Gram-positive bacteria 18, 19. In comparison to 31 *Pantoea* genomes recently scanned by BAGEL and antiSMASH, MMB-1 appears unique in generating hits for lankacidin C or bottromycin 15, 20.

In summary, the study here identified a strain of *Pantoea ananatis* (MMB-1) from soil, that appears capable of producing at least one antimicrobial substance, according to collected phenotypic and genotypic data. These findings increase our overall understanding of *Panteoa* as a group, and are also consistent with the notion that members of this group have significant potential as useful natural product producers, including as yet to be discovered antimicrobials. Future studies will seek to isolate and structurally-characterize anti-microbial products synthesized by MMB-1.
